# Protectin conjugates in tissue regeneration 1 restores lipopolysaccharide-induced pulmonary endothelial glycocalyx loss via ALX/SIRT1/NF-kappa B axis

**DOI:** 10.1186/s12931-021-01793-x

**Published:** 2021-07-03

**Authors:** Xin-Yang Wang, Xin-Yu Li, Cheng-Hua Wu, Yu Hao, Pan-Han Fu, Hong-Xia Mei, Fang Chen, Yu-Qiang Gong, Sheng-Wei Jin, Hui Li

**Affiliations:** Department of Anaesthesia and Critical Care, The Second Affiliated Hospital and Yuying Children’s Hospital of Wenzhou Medical University, 109 Xueyuan Road, Wenzhou, 325027 Zhejiang People’s Republic of China

**Keywords:** PCTR1, Glycocalyx, HPA, EXT-1, SIRT1

## Abstract

**Background:**

Endothelial glycocalyx loss is integral to increased pulmonary vascular permeability in sepsis-related acute lung injury. Protectin conjugates in tissue regeneration 1 (PCTR1) is a novel macrophage-derived lipid mediator exhibiting potential anti-inflammatory and pro-resolving benefits.

**Methods:**

PCTR1 was administrated intraperitoneally with 100 ng/mouse after lipopolysaccharide (LPS) challenged. Survival rate and lung function were used to evaluate the protective effects of PCTR1. Lung inflammation response was observed by morphology and inflammatory cytokines level. Endothelial glycocalyx and its related key enzymes were measured by immunofluorescence, ELISA, and Western blot. Afterward, related-pathways inhibitors were used to identify the mechanism of endothelial glycocalyx response to PCTR1 in mice and human umbilical vein endothelial cells (HUVECs) after LPS administration.

**Results:**

In vivo, we show that PCTR1 protects mice against lipopolysaccharide (LPS)-induced sepsis, as shown by enhanced the survival and pulmonary function, decreased the inflammatory response in lungs and peripheral levels of inflammatory cytokines such as tumor necrosis factor-α, interleukin-6, and interleukin-1β. Moreover, PCTR1 restored lung vascular glycocalyx and reduced serum heparin sulphate (HS), syndecan-1 (SDC-1), and hyaluronic acid (HA) levels. Furthermore, we found that PCTR1 downregulated heparanase (HPA) expression to inhibit glycocalyx degradation and upregulated exostosin-1 (EXT-1) protein expression to promote glycocalyx reconstitution. Besides, we observed that BAY11-7082 blocked glycocalyx loss induced by LPS in vivo and in vitro, and BOC-2 (ALX antagonist) or EX527 (SIRT1 inhibitor) abolished the restoration of HS in response to PCTR1.

**Conclusion:**

PCTR1 protects endothelial glycocalyx via ALX receptor by regulating SIRT1/NF-κB pathway, suggesting PCTR1 may be a significant therapeutic target for sepsis-related acute lung injury.

## Background

Sepsis is a common condition responsible for high morbidity and mortality worldwide [1]. Acute respiratory distress syndrome (ARDS), a severe sepsis complication, contributes to its high mortality [2]. Currently, there are no effective treatments or prevention available for sepsis-related lung injury [3–5]. The endothelium regulates the pathophysiology of lung injury [6]. The endothelial glycocalyx (EGL) primarily consists of glycosaminoglycans and proteoglycans, coating all healthy vascular endothelium [7]. Shedding of the endothelial glycocalyx is mainly driven by activation of heparan sulfate-specific heparanase, HPA [8]. Activated HPA promotes the degradation of the glycosaminoglycans and proteoglycans, and increases the level of glycocalyx components in the bloodstream, mainly including heparin sulphate (HS), hyaluronic acid (HA), and syndecan-1 (SDC-1) [8]. Exostosin-1 (EXT-1), a heparan sulfate elongation gene, is involved in glycocalyx synthesis and restitution, which is delayed in sepsis [9]. Damaged glycocalyx leads to an increase in permeability to proteins and fluids, causing interstitial leakage [10, 11]. Therefore, it is critical to improve glycocalyx reconstitution for the treatment of sepsis.

Protectin conjugates in tissue regeneration 1 (PCTR1) is a newly discovered member of the specialized pro-resolving mediators (SPMs) from docosahexaenoic acid (DHA) belonging to the peptide-conjugated Protectins [12, 13]. PCTR1 is identified and present in vivo before infection, and increases after LPS-induced inflammation, especially in inflammation resolution phrase [14]. Notably, PCTR1 not only enhances inflammation resolution by promoting macrophage efferocytosis and phagocytosis in infectious disease models but also improves tissue reparation [15, 16]. However, the effect of PCTR1 on endothelial glycocalyx is still undefined.

Herein, the effects of PCTR1 on LPS-induced inflammation were investigated using a murine model for sepsis. Specifically, we examined its impact on survival rate, lung function, and inflammatory injury in the lungs. Next, we investigated its effect on lung vascular endothelial glycocalyx in mice. To obtain a more well-defined observation, LPS-induced injury in human umbilical vein endothelial cells (HUVECs) was performed to determine the direct effect of PCTR1 on endothelial glycocalyx. Finally, we determined the effect of the receptor, as well as pathways targeted by PCTR1 on LPS-induced glycocalyx loss to gain insights into the underlying mechanisms.

## Methods

### Animal preparation

Specific pathogen-free (SPF) adult male C57BL/6 mice (6–8 wk) were bought from SLAC Laboratory Animal CO. (Shanghai, China). Before the experiments, the mice were kept in standard cages in a house controlled to a 12 h light/dark cycle with temperature maintained at 22–24 °C, humidity at 50–60% and SPF environment in Wenzhou Medical University. The mice were allowed free access to water and food. All animal procedures conformed to the Guide for the Care and Use of Laboratory Animals. This was approved by the Animal Studies Ethics Committee of Wenzhou Medical University.

### Animal experimental groups

Mice were intraperitoneally injected with LPS (15 mg/kg, Escherichia coli, serotype 055: B5; Sigma, Saint Louis, MO, USA) for survival experiments and LPS (10 mg/kg) for other experiments. The effect of PCTR1 (Cayman Chemical, Ann Arbor, MI, USA) on LPS-induced sepsis model was determined by establishing four experimental groups: control group, LPS group, LPS + PCTR1 group (LPS and PCTR1 were given simultaneously), PCTR1 group. For the PCTR1 and the control groups, the mice were intraperitoneally injected with PCTR1 100 ng/mouse or an equal saline volume. Next, to determine the mechanism of PCTR1, four groups were established: control group, LPS group, LPS + PCTR1 group, LPS + PCTR1 + BOC-2 group, LPS + PCTR1 + EX527 group, and LPS + BAY11-7082 group. Mice were administered with BOC-2 (600 ng/kg, Biomol-Enzo Life Sciences, Farmingdale, NY), EX527 (10 mg/kg, MEC, Shanghai, China), and BAY11-7082 (20 mg/kg, Selleck, Houston, TX, USA) intraperitoneally following LPS administration. The mice were anesthetized with 1% pentobarbital and sacrificed 6 h later. We obtained blood samples via the ophthalmic artery from mice that survived in each group, and obtained lung specimens.

### Cell culture and experimental groups

Human umbilical vein endothelial cells (HUVECs) were purchased from SGST (China). The cells were suspended in a DMEM medium containing 10% FBS and cultured in 25 cm^2^ flasks placed in an incubator controlled at 5% CO_2_ at 37 °C. In all experiments, HUVECs were added to the wells of six-well plates at equal concentrations and were further sub-divided into six groups: control group, LPS group, LPS + PCTR1 group (LPS and PCTR1 were given simultaneously), LPS + PCTR1 + DMSO group (DMSO was used as the solvent for EX527), LPS + PCTR1 + EX527 group and LPS + BAY11-7082 group. DMSO, EX527 (1 µM), and BAY11-7082 (1 µM) were added to cells for 24 h followed by LPS (1 µg/ml) and/or PCTR1 (100 nM) stimulation. The LPS or PCTR1 we used in vitro are the same one with we used in vivo.

### Invasive evaluation of respiratory mechanics

The lung function assay was conducted using a flexiVent system (Scireq, Montreal, QC, Canada), as described previously [17]. In brief, mice were anesthetized with 90 mg/kg pentobarbital sodium injected intraperitoneally and then tracheotomized. Vecuronium bromide was administered into each mice via intravenous injection, followed by mechanical ventilation using a computer-moderated small-animal ventilator. A model of deep inflation was used to record the inspiratory capacity (IC). Cst (quasistatic compliance) was assessed based on the PV curves.

### Lung morphology

Left lungs were excised after anesthetization, and fixed overnight with 4% paraformaldehyde at room temperature. This was followed by staining of 5-μm sections using hematoxylin and eosin (HE) and microscopic examination. The lung injury score was determined based on the level of infiltration of inflammatory cells, hyperemia, and the thickness of the alveolar wall.

### Lung vascular permeability assay

The evans blue dye (EBD) extravasation was utilized to assess pulmonary vascular permeability. Five and a half hours following LPS administration, EBD (20 mg/kg) was injected through the caudal vein. After the circulation of the dye for 30 min, perfusion of lungs was performed with saline (25 ml) under anaesthesia. Subsequently, the lungs were excised, blotted dry, weighed, and homogenized in formamide. After overnight extraction, the tissue fluid was centrifugated for 10 min at 12,000×*g*. A microplate reader was used to read the EBD concentration of the resultant supernatant at 620 nm absorbance.

### Wet-to-dry lung weight ratio

The index of pulmonary edema was determined from the wet-to-dry (W/D) lung weight ratio. Portions of the harvested wet left lungs were weighed and heated in an oven at 60 °C for 48 h. The W/D ratio was then determined after re-weighing the portions as dry weight.

### ELISA

Blood samples were obtained to determine level serum inflammatory cytokines, as well as the degradation products of glycocalyx in circulation. The blood was collected using the orbital sinus extraction method under anaesthesia. The serum was isolated from the blood samples for ELISA tests. Serum levels of cytokines including TNF-α, IL-1β, and IL-6 and glycocalyx-related proteins including HS, SDC-1, and HA were quantified using R&D systems and Boyun biotech ELISA kits following protocols provided by the manufacturer.

### Western blot

Lung tissues were homogenized with lysis buffer (RIPA: PMSF = 1:1) to obtain protein samples. The tissue homogenates were ultrasonicated and then centrifuged for 30 min at 12,000×*g*. The concentration of proteins in the supernatants was measured using the BCA kit. An equal amount of protein from each group was loaded onto 10% SDS-PAGE gels and resolved. The proteins were electro-transferred onto PVDF membranes. After being blocked with 10% milk for 2 h, membranes were incubated with the primary antibodies: EXT-1 (1:2000, GeneTex, Irvine, CA, USA), HPA (1:1000, Abcam, Cambridge, MA, USA), p65 (1:1000, Abcam, Cambridge, MA, USA), p-p65 (1:1000, Abcam, Cambridge, MA, US), SIRT1 (1:1000, Abcam, Cambridge, MA, USA), and β-actin (1:1000, BOYUN, Shanghai, China) overnight at 4 ℃. Next, the membranes were washed thrice and incubated with secondary antibody (1:3000, BOYUN, Shanghai, China) at room temperature for 1 h followed by another three-times wash. The protein bands were analyzed by Image Quant LAS 4000 mini (GE Healthcare Bio-Sciences AB, Uppsala, Sweden). The band intensity was analyzed with ImageJ.

### Immunofluorescence

Immunofluorescence was carried out using lung tissues and HUVECs. Lung sections were prepared for immunofluorescence after deparaffinized, dehydrated and antigen retrieval. This was followed by fixation of the HUVECs in 4% paraformaldehyde. Subsequently, the fixed cells and tissue samples were mounted on the cover glass, blocked with donkey serum (Solarbio, Beijing, China) and probed with HSPG2-antibody (1:200). After rinsing thrice with PBS, the fixed cells and tissue sections were incubated with the secondary antibody (1:200) at 37 ℃ for 1 h and further incubated with DAPI (Abcam) for 5 min. Eventually, both fixed cells and sectioned tissues were covered with an antifade mounting medium (Solarbio, Beijing, China) for fluorescence microscopy (Leica).

### Data analysis

The mean values are presented as the mean ± SD and were analyzed with GraphPad Prism 7.0 software. Mean values of groups were compared one-way ANOVA followed by Tukey’s post hoc test for multiple comparisons. Kaplan–Meier analysis was conducted to evaluate survival and a log-rank (Mantel-Cox) test was employed to determine statistical significance. *P* < 0.05 was considered statistically significant.

## Results

### PCTR1 improves survival and lung function in mice after LPS administration

**Fig. 1:**
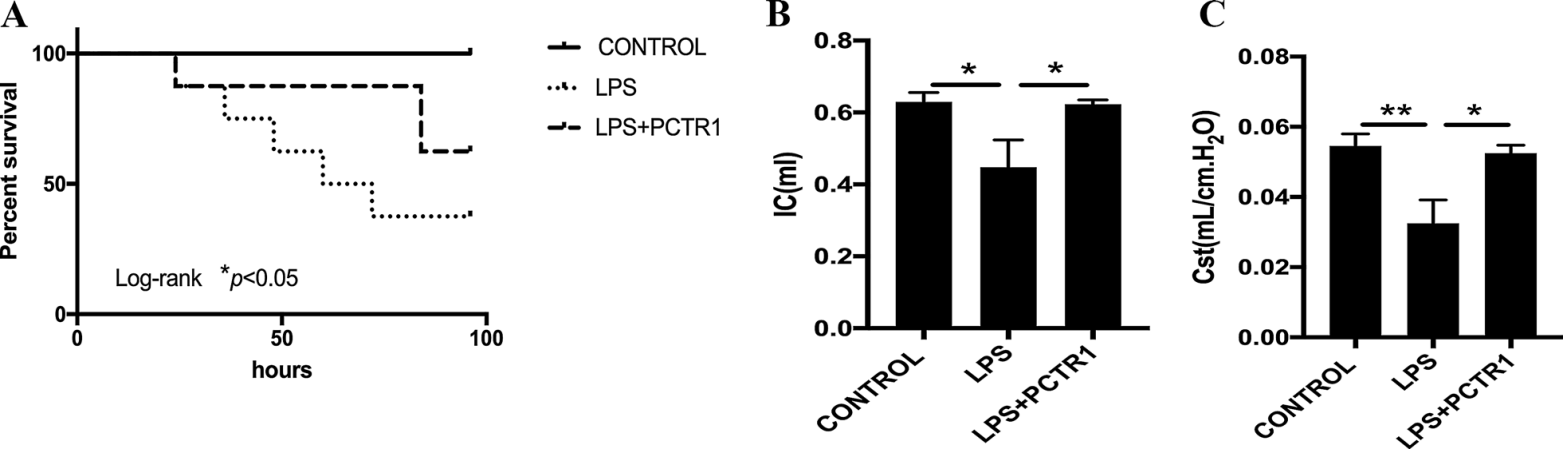
PCTR1 improves survival and lung function in mice after LPS administration. **a** Kaplan–Meier survival curves for the LPS (15 mg/kg) with or without PCTR1 (100 ng/mouse) treated mice (n = 8). Lung function of each group mice was measured by the flexiVent apparatus at 6 h after LPS (10 mg/kg) with or without PCTR1 (100 ng/mouse) challenge. **b**, **c** IC (Inspiratory Capacity) and Cst (Static Compliance) are shown (n = 5). *P < .05, **P < .01

We first determine the effect of PCTR1 on septic mice by survival curve analysis. PCTR1 significantly promoted survival in mice subjected to LPS (15 mg/kg) injected (Fig. 1a). Next, we performed a lung function test on LPS (10 mg/kg)-induced endotoxemia mice. Compared with the saline group, mice with LPS challenge showed a statistically significant reduction in IC and Cst, whereas PCTR1 enhanced IC and Cst significantly (Fig. 1b, c).

### PCTR1 alleviates LPS-stimulated lung inflammatory response in mice

**Fig. 2:**
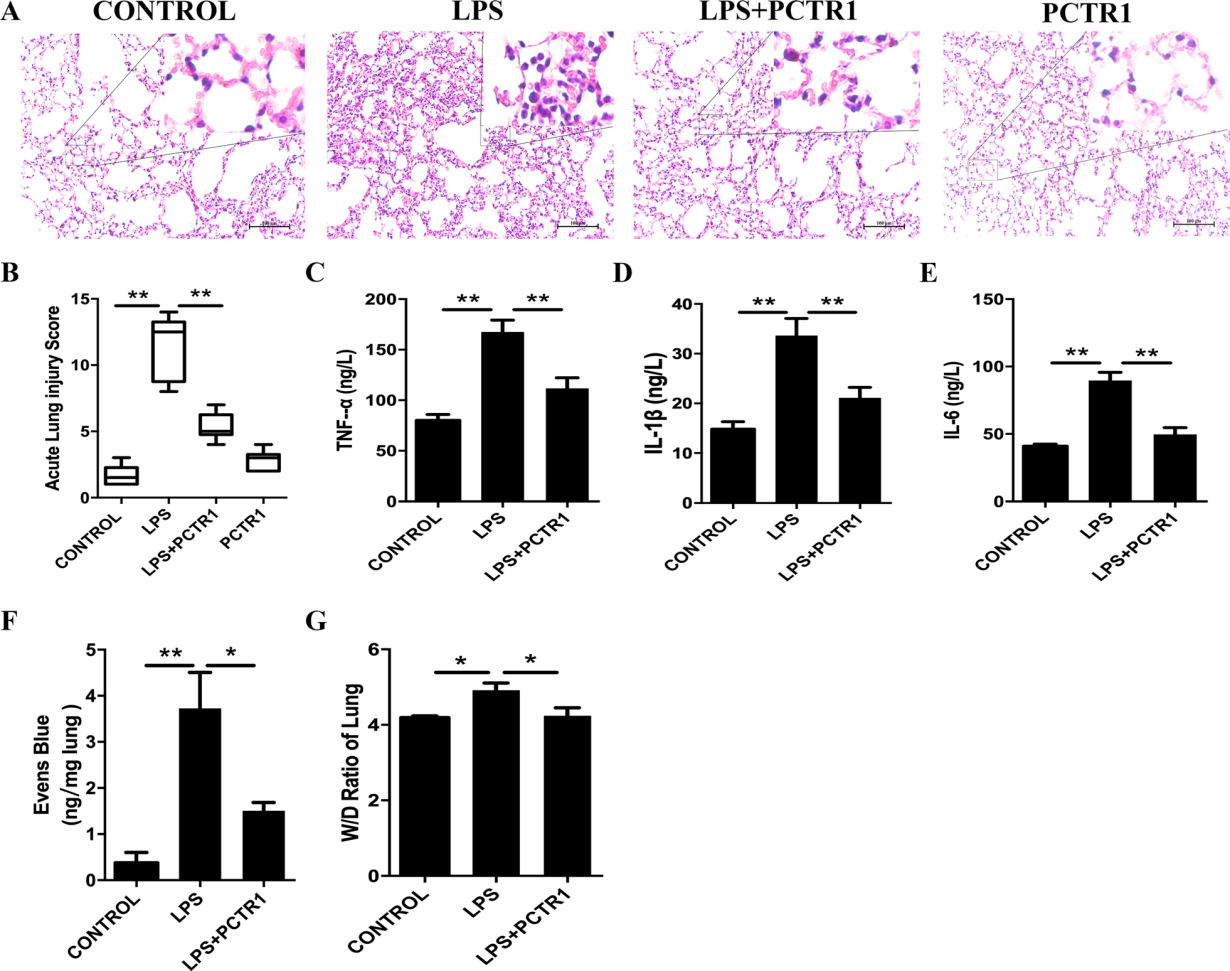
PCTR1 alleviates LPS-triggered lung inflammatory response in mice. Mice were treated with LPS (10 mg/kg) with or without PCTR1 (100 ng/mouse) for 6 h. **a** Representative images of Hematoxylin and eosin-stained in lung tissues are shown (X200). **b** Lung injury score (n = 6). **c**–**e**The serum levels of TNF-α, IL-1β and IL-6 were quantified using ELISA (n = 5). **f**, **g**Vascular permeability of lung tissues was determined using Evans Blue Dye and W/D weight ratio (n = 5 each). *P < .05, **P < .01

The histology in the control group showed normal lung conditions in mice. Notably, lung tissues in the LPS group were remarkably damaged, involving alveolar disarray, as well as infiltration level of inflammatory cells as indicated by increases in lung injury scores unlike mice in the control group. There was no marked change in morphologic features in the LPS + PCTR1 group. There was no remarkable difference between the control and the PCTR1 group (Fig. 2a, b). Accordingly, the peripheral levels of inflammatory cytokines, e.g., TNF-α, IL-1β, and IL-6 were remarkably upregulated in the LPS group relative to the control group and were lower in the PCTR1 treatment group compared with the LPS group (Fig. 2c–e). Furthermore, to determine pulmonary vascular permeability in mice, the EBD test and W/D ratio of lung tissues were assessed. As expected, PCTR1 significantly inhibited LPS induced EBD and W/D increase of lung tissues (Fig. 2f, g).

### PCTR1 restores LPS-induced endothelial glycocalyx damage in mice

**Fig. 3:**
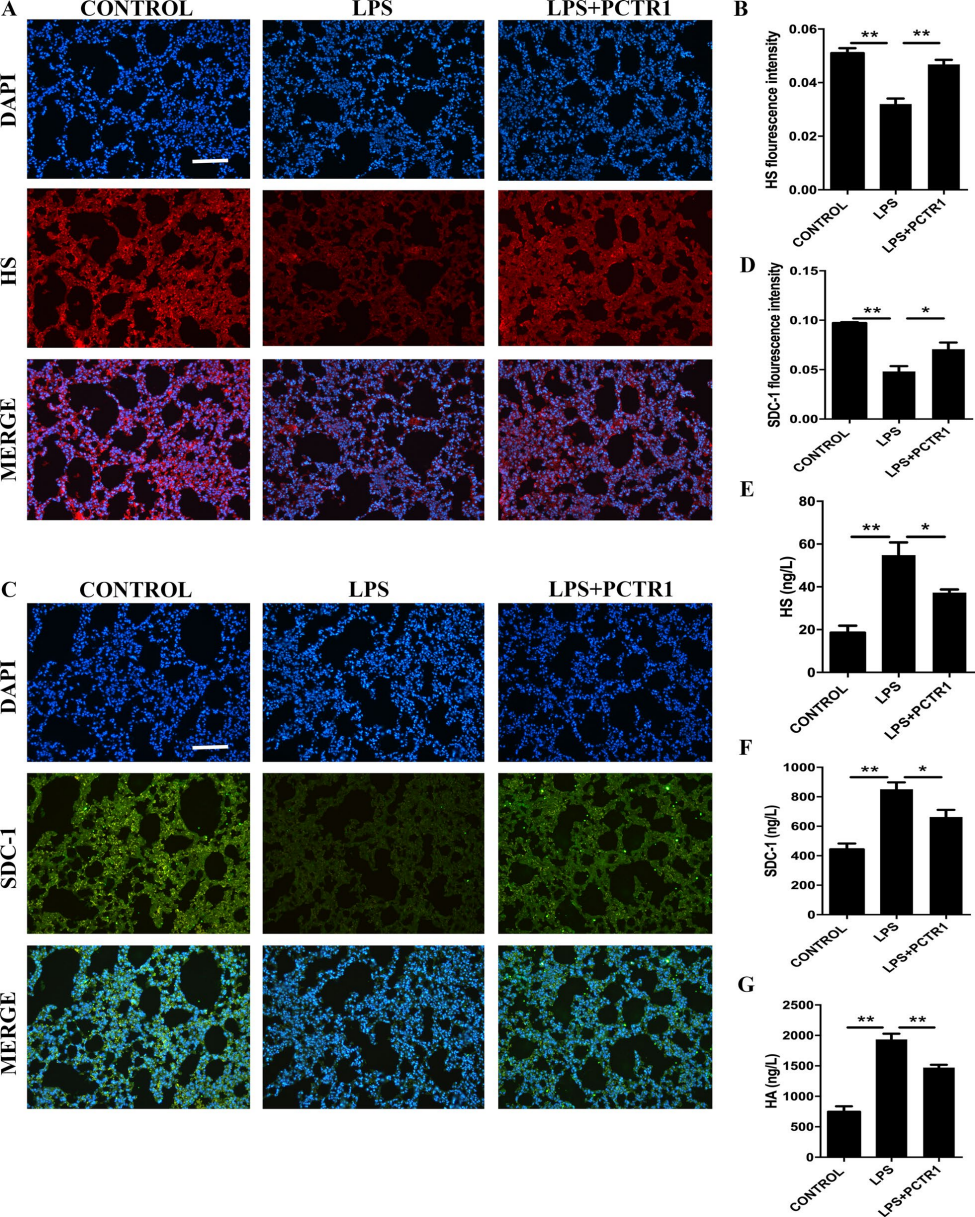
PCTR1 restores LPS-triggered endothelial glycocalyx damage in mice. LPS (10 mg/kg) with or without PCTR1 (100 ng/mouse) was injected to mice for 6 h. Representative image and densitometry of immunofluorescence of HS (**a**, **b**) and SDC-1 (**c**, **d**) in the lungs. Scar bar: 50 μm. **e**–**g** After collecting blood from the eyeballs, the serum levels of HS, SDC-1 and HA were quantified by ELISA. *P < .05, **P < .01, n = 5

On account of the importance of endothelial glycocalyx in maintaining vascular permeability, we tested the effect of PCTR1 on LPS-induced endothelial glycocalyx damage. Our results demonstrated that PCTR1increased the expression of HS and SDC-1 in lungs after LPS challenge (Fig. 3a–d). Accordingly, PCTR1 reduces the peripheral concentration of HS, SDC-1 and HA in septic mice (Fig. 3e–g).

### PCTR1 regulates HPA and EXT-1 expression in LPS-triggered sepsis in mice

**Fig. 4:**
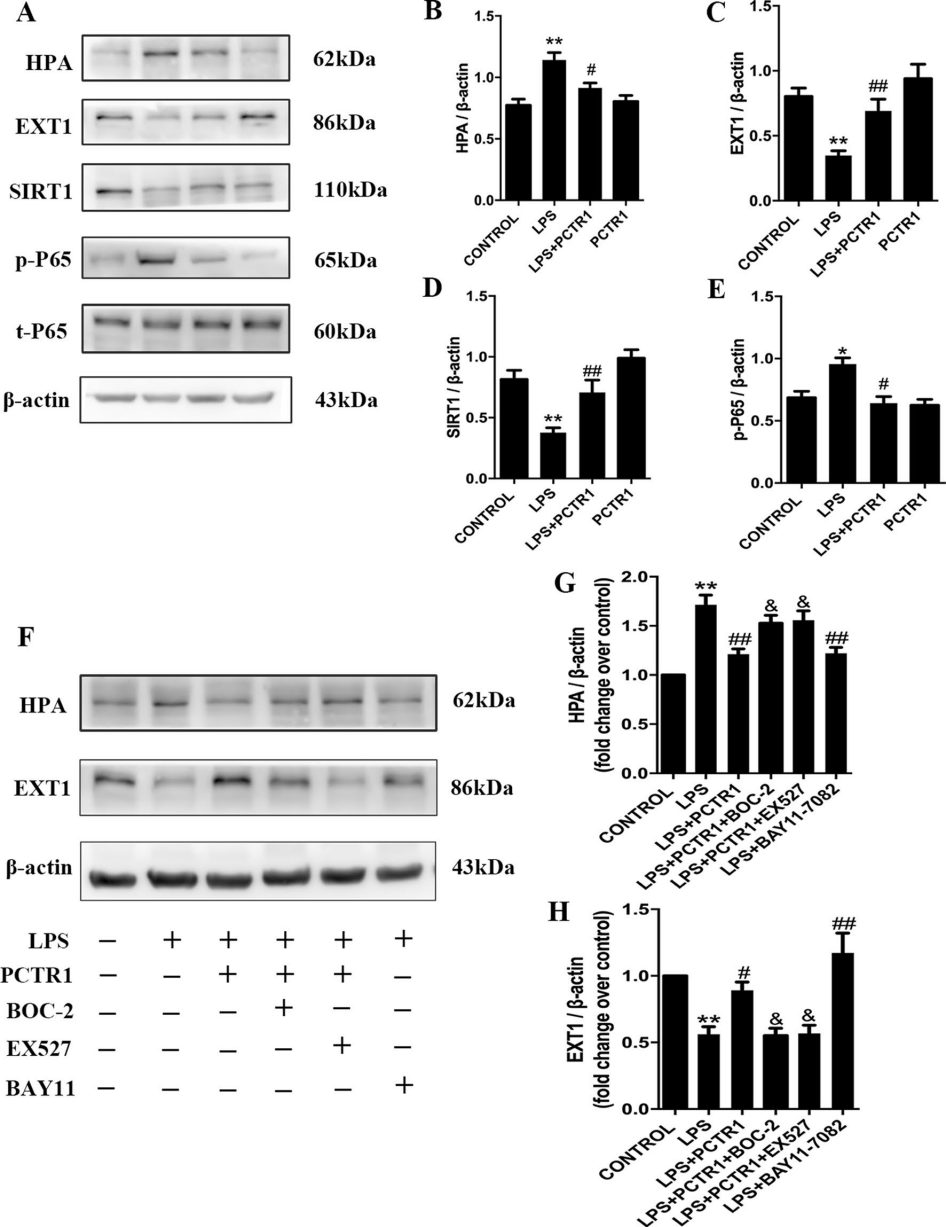
PCTR1 regulates HPA and EXT-1 expression in LPS- triggered sepsis in mice. **a**–**e** Mice were treated with LPS (10 mg/kg) with or without PCTR1 (100 ng/mouse) for 6 h. The levels of HPA, EXT-1, SIRT1 and p65 in the lung tissue were quantified by western blot. **f**–**h** BOC-2 (ALX receptor inhibitor, 600 ng/kg) or EX527 (SIRT1 inhibitor, 10 mg/kg) or BAY11-7082 (NF-κB inhibitor, 20 mg/kg) was injected 1 h followed by LPS (10 mg/kg) with or without PCTR1 (100 ng/mouse). The protein levels of HPA and EXT-1 were measured by western blot. *P < .05, **P < .01 relative to the CONTROL group, ^#^ *P* < .05, ^##^ *P* < .01 relative to the LPS group, ^&^*P* < .05, relative to the LPS + PCTR1 group. n = 6

The loss of endothelial glycocalyx induced pulmonary vascular hyperpermeability. HPA and EXT-1 are key enzymes involving in maintaining endothelial glycocalyx integrity via regulating degradation and reconstitution, respectively. PCTR1 decreased HPA expression and increased EXT-1 expression in LPS-induced septic mice. Meanwhile, the lower SIRT1 expression and its downstream, the higher NF-κB p65 phosphorylation (p-p65) expression were found in LPS group mice; by contrast, PCTR1 inhibited those changes in mice after LPS challenge (Fig. 4a, b). Together with the finding that, we postulate that PCTR1 might restore both HPA- and EXT-1-mediated endothelial glycocalyx dynamic balance through enhancing SIRT1 expression. As shown in our results, the inhibition of NF-κB blocked the effect of LPS on HPA and EXT-1, whilst the inhibition of SIRT1 blocked the effect of PCTR1 on HPA and EXT-1. Furthermore, we found that the effect of PCTR1 was abolished in the presence of BOC-2, the GFPR inhibitor (Fig. 4c, d).

### PCTR1 protects the endothelial glycocalyx by modulating SIRT1/NF-κB pathways via ALX receptor

**Fig. 5:**
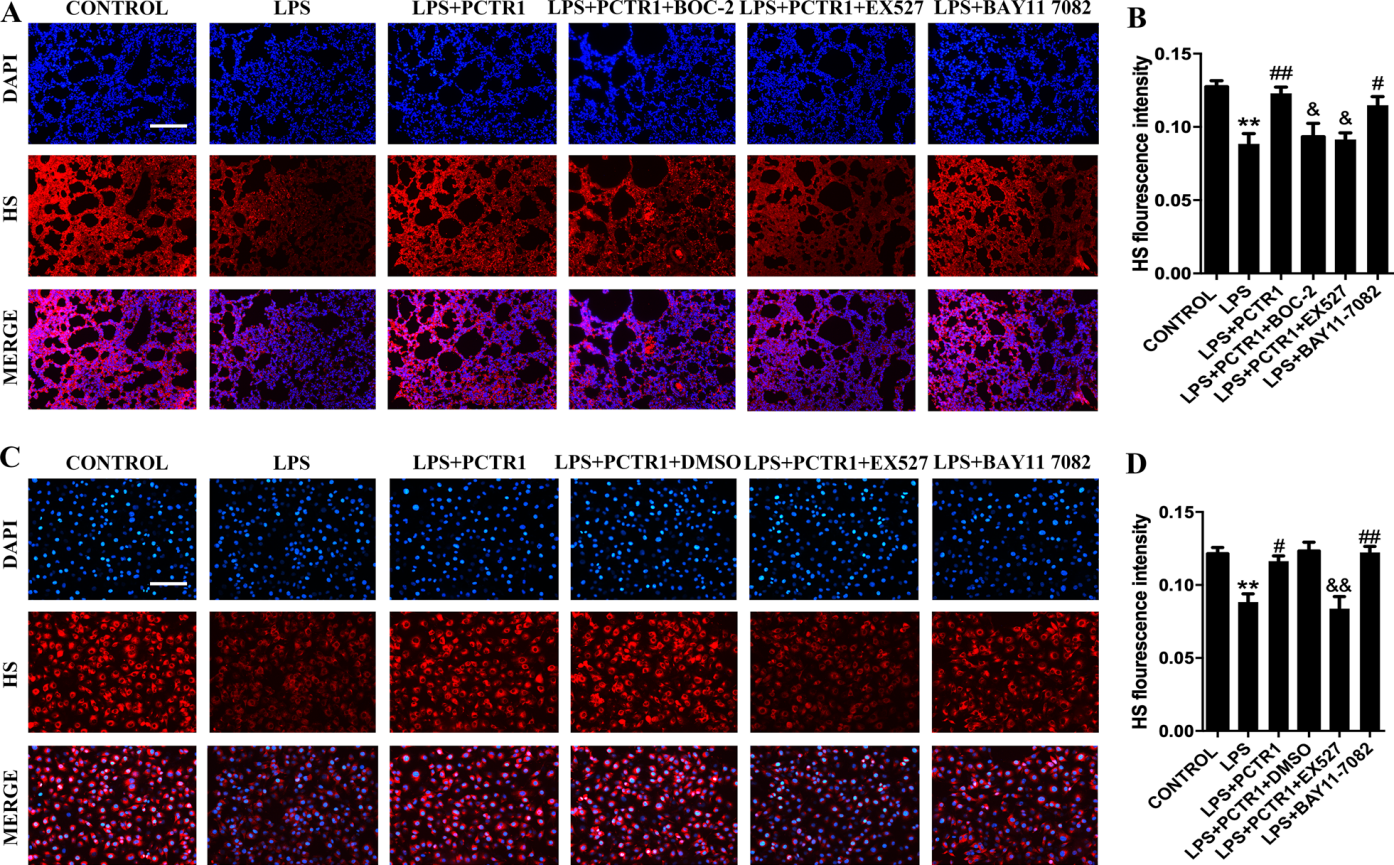
PCTR1 protects the endothelial glycocalyx by modulating SIRT1/NF-κB pathways via ALX receptor. The level of HPA, EXT-1, SIRT1 and p65 in lung tissues was quantified using western blotting assay. BAY11-7082 (NF-κB inhibitor, 20 mg/kg) or EX527 (SIRT1 inhibitor, 10 mg/kg) or BOC-2(ALX receptor inhibitor, 600 ng/kg) was injected 1 h followed by LPS (10 mg/kg) with or without PCTR1 (100 ng/mouse) for 6 h. (**a, b**) The expression of HS in tissue was examined by immunofluorescence. HUVECs were administered with 1 µg/ml LPS and 100 nM PCTR1 for 6 h. After treatment with EX527 or DMSO, HS in HUVECs was determined by immunofluorescence (**c, d**). Scar bar = 50 μm. **P < .01 in comparison to the CONTROL group, ^#^ *P* < .05, ^##^ *P* < .01 in comparison to the LPS group, ^&^*P* < .05, ^&&^*P* < .01, in comparison to the LPS + PCTR1 group. n = 6

To determine whether PCTR1 restores endothelial glycocalyx via ALX/SIRT1/NF-κB, we assessed the HS expression in lung sections of each group by immunofluorescence. As expected, the effect of LPS on HS was inhibited in the presence of BAY11-7082, and the effect of PCTR1 on HS was abolished in the presence of BOC-2 or EX527 (Fig. 5a, b). HUVEC endothelial cell was used for evaluating the effect of PCTR1 on endothelial glycocalyx in vitro. Consistent with the experiment results in vivo, we found that BAY11-7082 inhibited LPS-induced HS loss, and EX527 inhibited PCTR1-induced HS restoration in HUVECs after LPS stimulation (Fig. 5c, d).

## Discussion

**Fig. 6:**
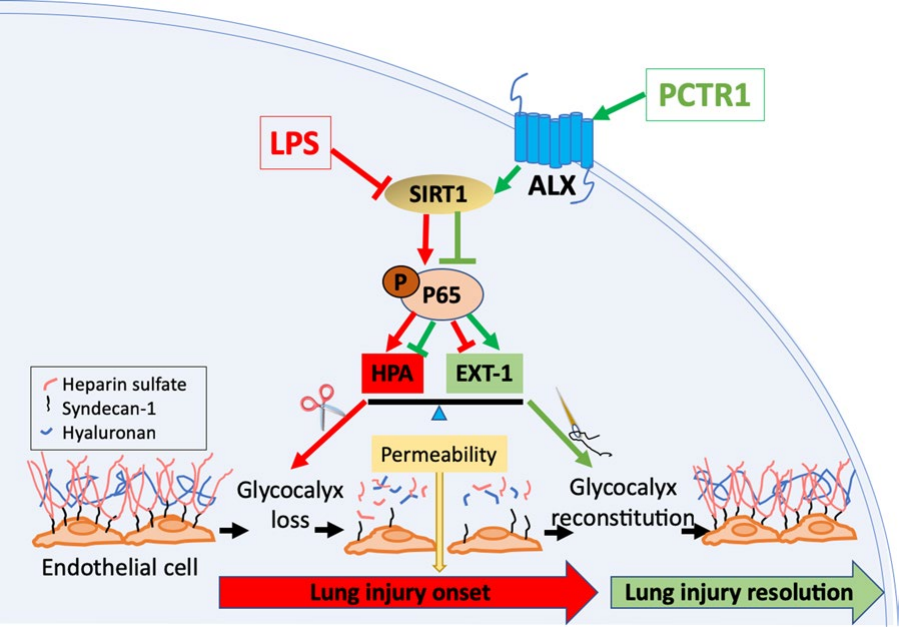
PCTR1 restores Lipopolysaccharide-induced pulmonary endothelial glycocalyx via ALX/SIRT1/NF-kappa B axis in mice. The effect of LPS was shown in red, whilst the effect of PCTR1 was shown in green. ALX: a G protein-coupled receptor; LPS: Lipopolysaccharides; PCTR1: Protectin conjugates in tissue regeneration 1; SIRT1: sirtuin 1; HPA: heparanase; EXT-1: exostosin-1

This study results provided research evidence of the protective effects of PCTR1 in a murine model of LPS injection. We first observed that PCTR1inhibited the infiltration of neutrophils, the generation of TNF-α, IL-1β, as well as IL-6 and LPS-provoked hyperpermeability in the lungs, with the outcome of enhanced survival and lung function. Next, we demonstrated that PCTR1 reduced lung endothelial glycocalyx degradation and improved glycocalyx reconstitution after LPS administration. Furthermore, we found PCTR1 decreased glycocalyx degradation factor HPA and increased glycocalyx key synthetase EXT-1 via ALX/SIRT1/NF-κB, restoring endothelial glycocalyx consequently (Fig. 6).

PCTR1 is a new endogenous mediator with the potential to promote inflammation resolution via G protein-coupled receptors(ALX receptor) [18]. It was demonstrated that PCTR1 is increased to enhance macrophage recruitment and phagocytosis during the inflammation resolution phase [14]. Moreover, PCTR1 generation in peritoneal lavage is decreased in vagotomized mice, suggesting that PCTR1 is controlled by local vagal to improve inflammation resolution [15]. Our previous studies demonstrated that PCTR1 reduced LPS-induced inflammation in mice via regulating linoleic acid metabolism, and improved pulmonary edema fluid clearance by activating the sodium channel and lymphatic drainage in LPS-triggered rat model [19, 20]. Similarly, we also observed that PCTR1 improved lung function and lung vascular leakage after LPS challenge in mice.

Increasing evidence demonstrates that the intact glycocalyx contributes to the maintenance of vascular integrity and leukocyte trafficking [21]. Whilst, after an acute injury, glycocalyx is degraded by HPA, and the level of glycocalyx components such as HS, SDC-1 and HA is elevated in the bloodstream, which could predict outcomes of patients with sepsis-induced organ dysfunction, including ARDS [22, 23]. In sepsis, EXT-1 expression is decreased, and glycocalyx reconstitution is delayed, which increases sepsis severity [24]. Previous studies has reported that enhancing endothelial fibroblast growth factor receptor 1 signaling improves EXT-1-mediated pulmonary glycocalyx reconstitution in sepsis [10, 25]. Thus, endothelial glycocalyx protection has been considered as a therapeutic strategy to treat sepsis. Herein, we found that PCTR1 unregulated EXT-1 expression and downregulated HPA expression, promoting glycocalyx reconstitution in sepsis model via ALX receptor consequently.

It has been demonstrated that SIRT1 is a negative regulator of inflammation [26]. Previous studies reported that endothelial glycocalyx decreased in vivo and in vitro with a defect in SIRT1 deacetylase activity [27, 28]. Consistently, we found that the protective effect of PCTR1 on glycocalyx was abolished in the presence of the inhibitor of SIRT1. SIRT1 has been reported to suppress inflammatory responses by inhibiting NF-κB signaling [29]. SIRT1 not only stimulates oxidative energy production but also inhibits transcription by deacetylating p65 and suppresses inflammation [30, 31]. On the other hand, the increases of HPA expression in various inflammation and cancer models with the increase of NF-κB P65 phosphorylation [32]. It is reported that NF-κB inhibitor attenuated HPA expression [32, 33]. In our study, we found that LPS increased HPA expression and decreased EXT-1 expression via activating NF-κB, degrading glycocalyx consequently in vivo. HUVEC was considered as a common choice for the endothelial experiment in vitro [34]. Consisitent results were also obtained from the experiment in HUVEC. Whilst, PCTR1 induced SIRT1 expression and reduced NF-κB phosphorylation after LPS administration and reversed the effect of LPS on glycocalyx in vivo and in vitro.

## Conclusions

This study indicates that PCTR1 reduces endothelial glycocalyx loss and improves glycocalyx reconstitution by modulating glycocalyx key enzymes including HPA and EXT-1 through ALX/SIRT1/NF-κB cascade after LPS treatment. The preservation of the glycocalyx attenuates lung inflammation response and vascular endothelial hyperpermeability, and further restores lung function and survival rate after LPS administration in mice. This study not only identifies a protective action of PCTR1 in LPS-induced sepsis mice, but the results may also open a new therapeutic strategy with PCTR1 for other vascular-related diseases.

## Data Availability

All supporting data are available from the corresponding author upon reasonable request.

## Abbreviations

PCTR1: Protectin conjugates in tissue regeneration 1
LPS: Lipopolysaccharide
HS: Heparin sulphate
SDC-1: Syndecan-1
HA: Hyaluronic acid
HPA: Heparanase
EXT-1: Exostosin-1
ARDS: Acute respiratory distress syndrome
EGL: Endothelial glycocalyx
SPMs: Specialized pro-resolving mediators
DHA: Docosahexaenoic acid
SPF: Specific pathogen-free
HUVECs: Human umbilical vein endothelial cells
IC: Inspiratory capacity
Cst: Quasistatic compliance
HE: Hematoxylin and eosin
EBD: Evans blue dye
W/D: Wet-to-dry
GPCR: G protein-coupled receptors
SIRT1: Sirtuin 1
